# The Mo–Se–Fe–NOS Signature: Linking trace Element Dysregulation to Endothelial Dysfunction in Gestational Diabetes Mellitus

**DOI:** 10.1007/s12011-026-05033-5

**Published:** 2026-02-21

**Authors:** Rıza Dur, Aycan Baş, Gamze Dur

**Affiliations:** 1 School of Medicine, Department of Obstetrics and Gynecology, Afyonkarahisar Health Sciences University, Zafer Sağlık Külliyesi Dörtyol Mahallesi 2078 Sokak No: 3, Afyonkarahisar, Turkey; 2 School of Medicine, Department of Biophysics, Afyonkarahisar Health Sciences University, Afyonkarahisar, Turkey; 3 School of Medicine, Department of Family Medicine, Afyonkarahisar Health Sciences University, Afyonkarahisar, Turkey

**Keywords:** Gestational diabetes mellitus, Trace elements, Nitric oxide, Endothelial dysfunction, Molybdenum, Biomarkers

## Abstract

Objective: Gestational diabetes mellitus (GDM) is a multifactorial metabolic disorder in which redox imbalance and endothelial dysfunction may contribute to adverse maternal–fetal outcomes. We investigated whether maternal serum essential and toxic element profiles differ in GDM and whether these alterations are associated with oxidative stress indices and nitric oxide (NO) bioavailability. Methods: Serum samples from 50 women with GDM and 50 healthy pregnant controls were analyzed. Essential trace elements (Zn, Cu, Se, Fe, Mn, Cr, Mo, V) and toxic metals (Pb, Cd, As, Sn, Co) were quantified using inductively coupled plasma mass spectrometry (ICP–MS). Total antioxidant status (TAS), total oxidant status (TOS), asymmetric dimethylarginine (ADMA), and nitric oxide synthase (NOS)-related NO metabolites were measured by ELISA-based methods. Results: NOS levels were significantly reduced in GDM (p < 0.001), while TAS, TOS, and ADMA did not differ between groups. Zn, Cu, Se, Fe, Mo, and V were significantly higher in GDM, whereas toxic metals showed no group differences. In exploratory ROC analyses, selenium showed the highest discrimination for GDM (AUC = 0.754, p < 0.001), followed by iron (AUC = 0.714, p < 0.001) and vanadium (AUC = 0.700, p = 0.001). Molybdenum demonstrated modest discrimination (AUC = 0.662, p = 0.005), indicating potential—rather than strong stand-alone—predictive value. In logistic regression (per 1 SD increase), selenium and molybdenum were associated with higher odds of GDM in age-adjusted models, supporting the relevance of the multi-marker profile. Conclusion: The co-occurrence of elevated redox-active elements with reduced NO bioavailability in GDM supports a hypothesis-generating “Mo–Se–Fe–NOS signature” (an observational multi-marker profile) that warrants longitudinal and external validation to clarify mechanisms and clinical utility.

**Supplementary Information:**

The online version contains supplementary material available at 10.1007/s12011-026-05033-5.

## Introduction

Gestational diabetes mellitus (GDM) is one of the most common metabolic disorders in pregnancy, characterized by glucose intolerance first recognized during gestation [1, 2]. Its prevalence continues to increase globally, paralleling the rise in obesity and maternal age [2, 3]. According to the International Diabetes Federation (IDF) and the American Diabetes Association (ADA), GDM affects up to 15–20% of all pregnancies, representing a major public health burden with long-term metabolic consequences for both mother and child [1, 2, 4].

Beyond hyperglycemia, GDM is now recognized as a multifactorial condition involving oxidative stress, endothelial dysfunction, inflammation, and dysregulated trace element metabolism [3–6]. Physiologically, pregnancy is a state of enhanced insulin resistance, oxidative load, and altered redox signaling [4, 5]. When adaptive mechanisms fail, increased production of reactive oxygen species (ROS) and impaired antioxidant defenses lead to oxidative damage to lipids, proteins, and DNA [7]. This redox imbalance contributes to endothelial nitric oxide synthase (eNOS) dysfunction, impaired nitric oxide (NO) bioavailability, and vascular inflammation—hallmarks of GDM-related endothelial injury [7, 8].

Trace elements play a crucial role in maintaining redox and metabolic homeostasis. Essential elements such as selenium (Se), zinc (Zn), copper (Cu), iron (Fe), manganese (Mn), chromium (Cr), and molybdenum (Mo) act as cofactors for antioxidant enzymes including superoxide dismutase, glutathione peroxidase, and catalase, thereby regulating oxidative stress and insulin sensitivity [7–9]. Disturbances in these elements—either deficiency or excess can alter mitochondrial metabolism, impair NO synthesis, and disrupt insulin signaling [9–11].

Briefly, Zn contributes to insulin synthesis, storage, and signaling and is a structural component of antioxidant defenses, while Cu is required for redox enzymes but, in excess, may promote pro-oxidant activity. Se supports selenoproteins (e.g., glutathione peroxidases, thioredoxin reductases) that regulate redox balance, yet supraphysiological Se may adversely affect metabolic and endothelial pathways. Fe is essential for oxygen transport and mitochondrial function but can catalyze ROS generation via Fenton chemistry, potentially impairing endothelial NO bioavailability. Mn and Cr have been implicated in antioxidant enzyme activity and insulin sensitivity, respectively, although findings in pregnancy are inconsistent. Mo, a cofactor for molybdoenzymes (e.g., xanthine oxidase), may influence oxidative stress pathways relevant to NO signaling, while V has insulin-mimetic properties and may modulate glucose metabolism. Given the growing literature on combined metal exposures, we also quantified selected toxic metals to explore whether non-essential elements contribute to the GDM biochemical milieu [7–12].

Recent studies have demonstrated that maternal serum concentrations of redox-active trace elements are significantly altered in GDM [9–11]. Elevated levels of Se, Cu, Fe, and Zn have been associated with increased oxidative stress and insulin resistance, whereas excessive Mo exposure may interfere with sulfur- and iron-dependent enzymes, amplifying metabolic dysregulation [11, 12]. In a large prospective cohort, multi-metal exposure involving Cu, Zn, and Cd during early pregnancy was linked to higher GDM risk, highlighting a dose-dependent synergistic effect between essential and toxic elements [10, 11].

Endothelial dysfunction further integrates these metabolic and oxidative mechanisms. Impaired NO synthesis, reflected by reduced NOS activity and elevated asymmetric dimethylarginine (ADMA), has been observed in women with GDM and correlates with poor vascular reactivity [8, 9, 13]. Structural and molecular studies in placental tissue confirm that GDM is characterized by altered endothelial signaling, reduced vasorelaxation, and increased oxidative burden [9, 13]. These mechanisms suggest that GDM is not solely a carbohydrate metabolism disorder but also a state of trace element induced vascular oxidative stress [9, 13].

Collectively, the current evidence underscores that GDM pathophysiology involves an intricate interplay between trace element imbalance, oxidative stress, and endothelial dysfunction [7, 9–13]. Understanding how redox-active trace elements influence oxidative and vascular pathways may yield novel biomarkers for early diagnosis and therapeutic targets for intervention. Therefore, the present study aimed to assess serum concentrations of essential and toxic trace elements, oxidative stress markers, and endothelial parameters in women with GDM compared to healthy pregnant controls, exploring potential mechanistic and predictive associations.

## Materials and Methods

This prospective case–control study was conducted to investigate the relationship between serum trace element concentrations, oxidative stress parameters, and endothelial function markers in women diagnosed with gestational diabetes mellitus (GDM) and healthy pregnant controls. A statistical power analysis based on the observed effect size for the primary outcome, nitric oxide synthase (NOS) activity (mean difference = 80.5 µmol/L, SD = 110.2), confirmed the robustness of the sample size. With *n* = 50 per group and α = 0.05, the study achieved a statistical power (1 − β) of 0.87, indicating adequate sensitivity to detect significant differences. Furthermore, for trace elements showing moderate differences (Cohen’s d ≈ 0.5), the calculated power exceeded 0.80, confirming sufficient sample size for the study objectives. The study included a total of 100 participants: 50 women with GDM and 50 age- and gestational age-matched healthy controls. Diagnosis of GDM was established between the 24th and 28th weeks of gestation based on the criteria of the American Diabetes Association (ADA, 2024) using a two-step oral glucose tolerance test [1]. Crucially, blood samples for the GDM group were collected immediately following the diagnosis and prior to the initiation of any therapeutic intervention, including dietary modification or insulin/pharmacological treatment, to assess the baseline pathological state. Healthy controls were defined as women with normal glucose tolerance, no pre-existing diabetes, and no history of hypertensive or metabolic disorders. Exclusion criteria included multiple pregnancies, chronic renal or hepatic disease, autoimmune or thyroid disorders, and the use of mineral or vitamin supplements. All participants provided written informed consent, and the study protocol was approved by the Institutional Ethics Committee in accordance with the Declaration of Helsinki. This case-control study was approved by the local ethics committee (approval no: 2022/12–498) and supported by Afyonkarahisar Health Sciences University Scientific Research Projects Coordination Unit (project no: 22.GENEL.047).

Venous blood samples were collected from each participant after overnight fasting. Sampling was performed between 08:00 and 10:00 a.m. from the antecubital vein into trace element–free vacutainer tubes. Samples were allowed to clot for 30 min and subsequently centrifuged at 3000 rpm for 10 min at 4 °C. The serum supernatant was separated into pre-labeled polypropylene tubes and stored at − 80 °C until analysis. All glassware and containers were acid-washed and rinsed with ultrapure water to prevent contamination. Hemolyzed samples were excluded from the study.

Serum concentrations of essential (Zn, Cu, Se, Fe, Mn, Cr, Mo, V) and toxic elements (Pb, Cd, As, Sn) were quantified using inductively coupled plasma mass spectrometry (ICP–MS, Agilent 7900, USA). Calibration was performed using multi-element standard solutions (High-Purity Standards, Charleston, USA), and quality control was ensured with certified reference materials (Seronorm Trace Elements Serum L-1 and L-2, Norway). Analytical precision and accuracy were verified by repeated measurements of control samples (coefficient of variation < 5%). Internal standards (rhodium and indium) were used for instrumental drift correction. All results were expressed in µg/L [12].

Serum total antioxidant status (TAS), total oxidant status (TOS), asymmetric dimethylarginine (ADMA), and nitric oxide synthase (NOS) activity were measured using commercially available enzyme-linked immunosorbent assay (ELISA) kits (Rel Assay Diagnostics, Gaziantep, Turkey). TAS and TOS levels were assessed colorimetrically according to the method described by Erel [14], providing an integrated estimate of total antioxidant capacity and total oxidant burden in serum. TAS reflects the cumulative contribution of serum antioxidants (including both enzymatic and non-enzymatic components) and therefore does not quantify individual antioxidants. Similarly, TOS represents a composite index of total oxidants and does not specify the contribution of particular reactive species. NOS activity was indirectly quantified by measuring the stable end-products of Nitric Oxide (NO) metabolism (nitrite and nitrate) via the Griess method integrated into the ELISA kit, thus reflecting circulating NO bioavailability. NOS and ADMA were quantified according to the manufacturer’s instructions, with intra- and inter-assay coefficients of variation below 8%. All measurements were performed in duplicate, and mean values were used for statistical analysis.

Statistical analyses were performed using SPSS software (Version 25.0, IBM Corp., Armonk, NY, USA). Data distribution was evaluated using the Kolmogorov–Smirnov test. Continuous variables were expressed as mean ± standard deviation (SD) or median (interquartile range) as appropriate. Group comparisons were made using Student’s t-test for normally distributed data and the Mann–Whitney U test for non-parametric data. Categorical variables were compared using the Chi-square test. Pearson or Spearman correlation analyses were used to evaluate associations between biochemical parameters and trace element concentrations. Receiver operating characteristic (ROC) curve analysis was performed to determine the diagnostic performance of trace elements and oxidative markers in predicting GDM, and the area under the curve (AUC) values were calculated. To control for the increased risk of Type I error arising from multiple comparisons involving 18 distinct element and biomarker measurements, the Benjamini–Hochberg False Discovery Rate (FDR) method was applied, with a corrected p-value reported where appropriate. A p-value < 0.05 was considered statistically significant. ROC analyses were performed for exploratory discrimination assessment and were not intended as definitive clinical prediction models. Additionally, binary logistic regression was performed with GDM status as the outcome. Trace elements were standardized (per 1 SD increase) to enable direct comparability across biomarkers. We report odds ratios (ORs) with 95% confidence intervals (CIs) for unadjusted models and age-adjusted models. Given the sample size, models were kept parsimonious. A two-sided p value < 0.05 was considered statistically significant.

## Results

The study included a total of 100 pregnant women, consisting of 50 patients with gestational diabetes mellitus (GDM) and 50 healthy pregnant controls. No significant differences were observed between the groups in terms of age, gestational age, or body mass index (BMI).

Serum concentrations of redox-active trace elements, including zinc (Zn), copper (Cu), selenium (Se), iron (Fe), molybdenum (Mo), and vanadium (V) were compared between the GDM and control groups (Table 1). Levels of Zn (*p* = 0.006), Cu (*p* = 0.001), Se (*p* < 0.001), Fe (*p* < 0.001), Mo (*p* = 0.006), and V (*p* < 0.001) were significantly higher in the GDM group compared to the controls. These differences remained significant after applying the Benjamini–Hochberg False Discovery Rate correction. Manganese (Mn) and Chromium (Cr) levels did not differ significantly.

**Table 1:** Serum trace element concentrations in healthy pregnant women and women with GDM (median (IQR))

Element	Healthy pregnant median (IQR)	Women with GDM median (IQR)	*p*
Zinc (Zn)	65.0 (24.5)	78.5 (24.8)	0.006
Copper (Cu)	161.0 (57.0)	188.5 (73.3)	0.001
Selenium (Se)	40.0 (19.0)	52.5 (20.8)	< 0.001
Iron (Fe)	69.0 (56.5)	105.5 (64.0)	< 0.001
Chromium (Cr)	1.3 (2.1)	1.1 (1.3)	0.878
Vanadium (V)	0.6 (0.3)	0.8 (0.3)	< 0.001
Molybdenum (Mo)	1.1 (0.5)	1.4 (0.6)	0.006

The comparison of non-essential (toxic metal) trace element concentrations (lead [Pb], cadmium [Cd], arsenic [As], cobalt [Co] and tin [Sn]) is summarized in Table 2. No statistically significant differences were found between GDM and control groups for any of these elements (*p* > 0.05).

**Table 2:** Serum heavy metal concentrations in healthy pregnant women and women with GDM (median (IQR))

Element	Healthy pregnant median (IQR)	Women with GDM median (IQR)	*p*
Cadmium (Cd)	0.1 (0.0)	0.1 (0.0)	0.689
Lead (Pb)	0.1 (0.1)	0.1 (0.1)	0.506
Manganese (Mn)	1.9 (1.3)	2.0 (1.9)	0.197
Tin (Sn)	0.4 (0.2)	0.3 (0.4)	0.128
Arsenic (As)	0.3 (0.4)	0.4 (0.4)	0.374

Oxidative stress and endothelial function markers were evaluated (Table 3). There were no significant differences in Total Antioxidant Status (TAS), Total Oxidant Status (TOS), or Asymmetric Dimethylarginine (ADMA) levels between groups (*p* > 0.05). However, NOS activity was significantly lower in the GDM group (Median: 51.9 µmol/L) compared with the control group (Median: 132.4 µmol/L) (*p* < 0.001).

**Table 3:** Comparison of oxidative stress parameters and endothelial markers between groups (median (IQR))

Parameter	Healthy pregnant median (IQR)	Women with GDM median (IQR)	*p*
TAS (U/mL)	12.1 (19.6)	11.7 (9.9)	0.421
TOS (U/mL)	6.1 (11.9)	6.7 (7.1)	0.997
ADMA (ng/L)	8720.6 (21493.6)	11861.6 (15126.1)	0.062
NOS (µmol/L)	132.4 (277.3)	51.9 (91.3)	< 0.001

Correlation analyses within the GDM group are summarized in Tables 4 and 5. ADMA showed a significant inverse correlation with Zn (*r* = − 0.432, *p* = 0.003). NOS was inversely correlated with Zn (*r* = − 0.337, *p* = 0.025) and Cu (*r* = − 0.307, *p* = 0.042), while other correlations were not statistically significant. These correlations should be interpreted as exploratory.

**Table 4:** Correlation between oxidative stress parameters and trace elements in women with GDM (Spearman’s r)

TAS (*r*)	Zn	Cu	Se	Fe	Cr	V	Mo
	-0.203	-0.194	-0.060	0.015	-0.192	-0.029	0.085
TAS (p)	0.186	0.207	0.698	0.924	0.211	0.852	0.582
TOS (r)	-0.172	-0.106	0.213	-0.157	-0.206	0.107	0.189
TOS (p)	0.264	0.492	0.166	0.308	0.179	0.487	0.219
ADMA (r)	-0.432	0.007	-0.094	-0.061	-0.203	0.152	0.217
ADMA (p)	0.003	0.962	0.545	0.693	0.185	0.324	0.157
NOS (r)	-0.337	-0.307	-0.191	0.007	-0.289	0.039	0.087
NOS (p)	0.025	0.042	0.213	0.966	0.057	0.802	0.574

**Table 5:** Correlation between oxidative stress parameters and heavy metals in women with GDM (Spearman’s r)

TAS (*r*)	Cd	Pb	Mn	Sn	As
	-0.091	0.159	0.240	-0.010	0.231
TAS (p)	0.557	0.303	0.117	0.950	0.132
TOS (r)	0.091	-0.090	0.082	-0.114	0.141
TOS (p)	0.558	0.560	0.596	0.462	0.360
ADMA (r)	-0.168	0.124	0.152	-0.050	0.104
ADMA (p)	0.276	0.422	0.324	0.747	0.503
NOS (r)	-0.138	0.051	0.049	-0.037	0.071
NOS (p)	0.372	0.740	0.751	0.810	0.645

**Fig. 1:**
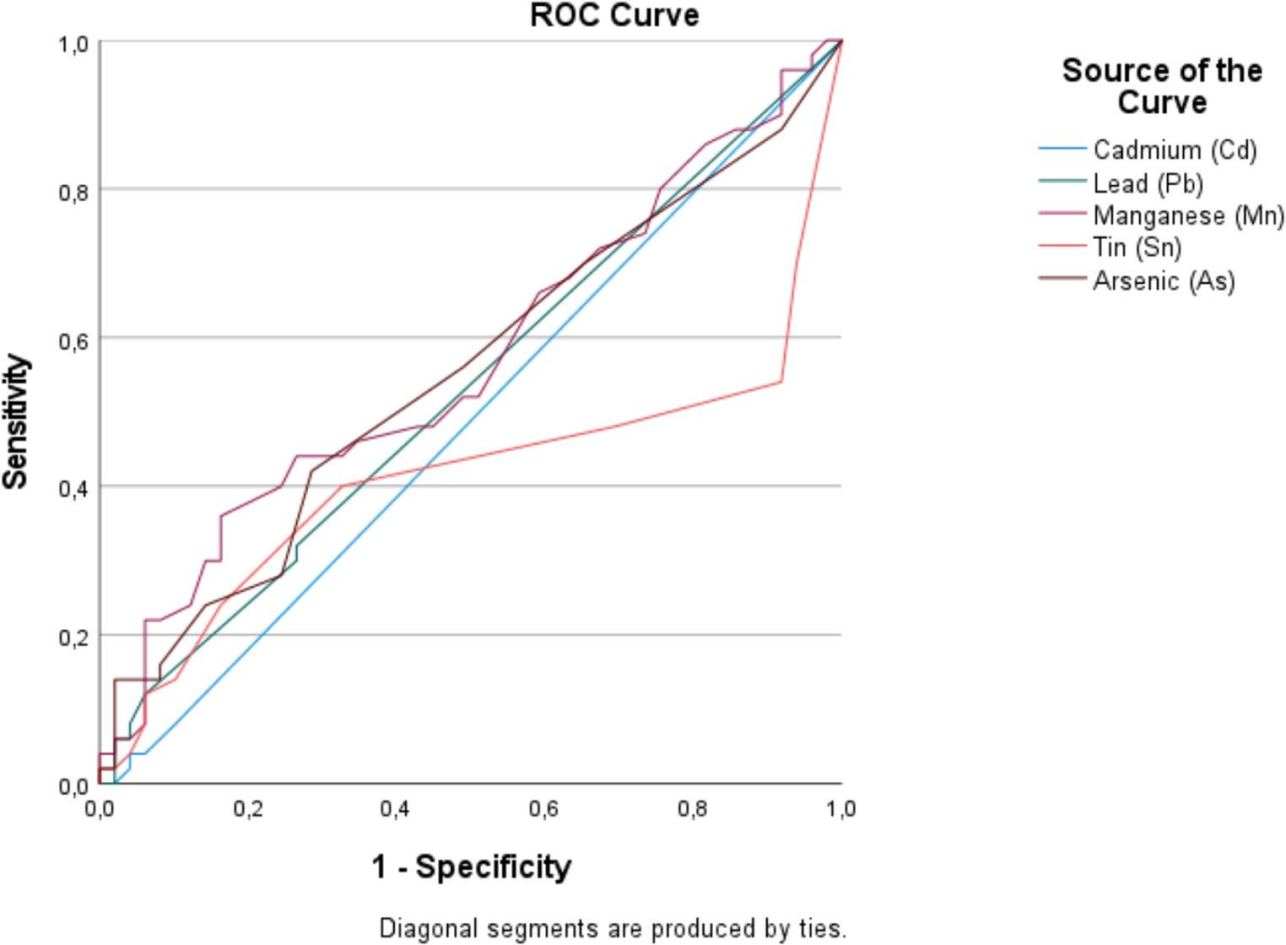
ROC curves for toxic metals

**Fig. 2:**
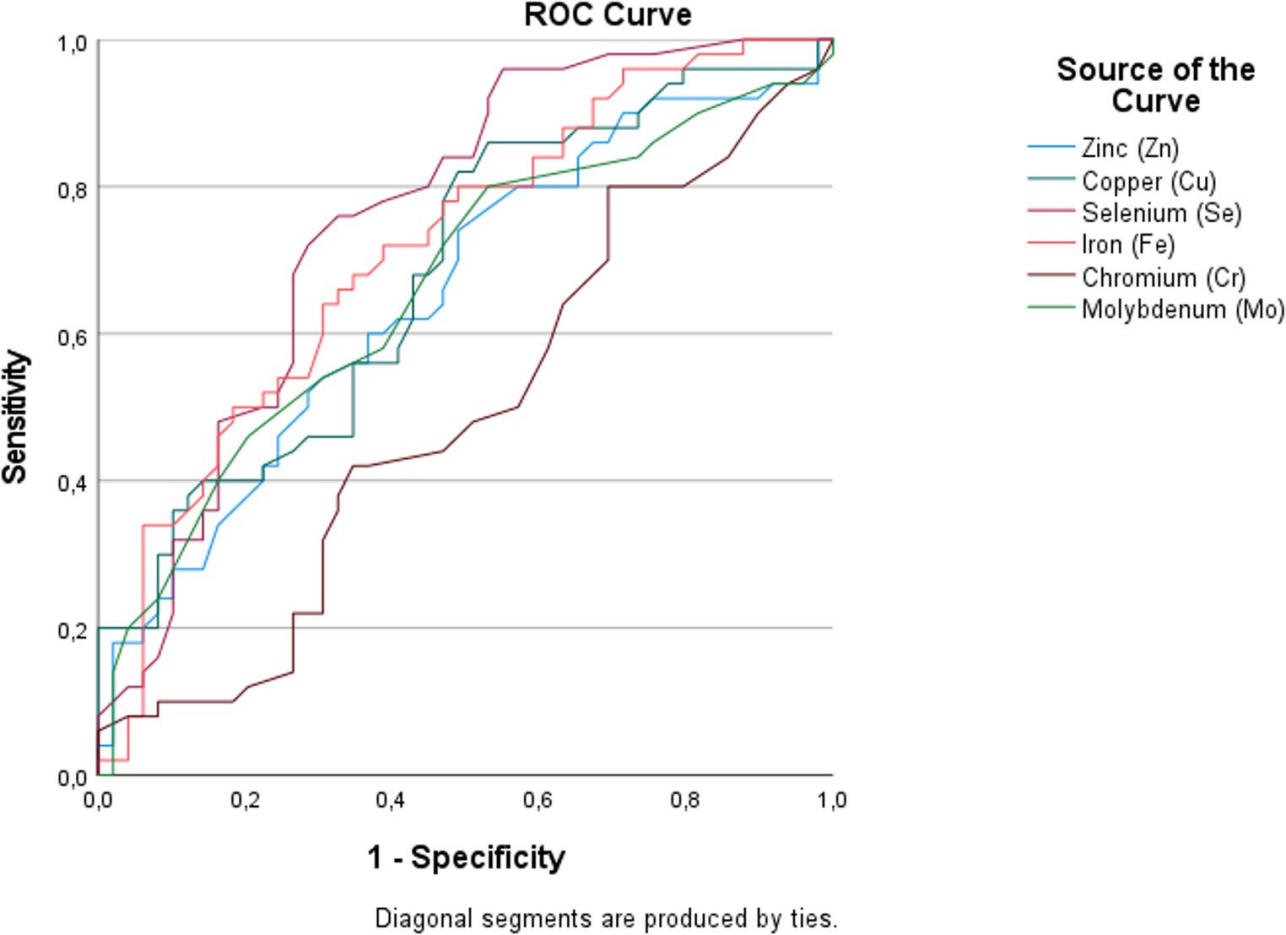
ROC curves for essential trace elements

Receiver operating characteristic (ROC) analyses were performed to assess the diagnostic performance of individual trace elements in predicting GDM (Table 6). Selenium (Se) exhibited the highest diagnostic accuracy with an AUC of 0.754(*p* < 0.001). At the optimal cut-off value of 46.5 µg/L, Se demonstrated a sensitivity of 72.0% and a specificity of 71.4% for predicting GDM. Iron (Fe, AUC 0.714) and Vanadium (V, AUC 0.700) also showed high discriminatory ability. Molybdenum (Mo) reached an AUC of 0.662 (*p* = 0.005), notably demonstrating a high sensitivity of 80.0%(Specificity: 46.9%) at its optimal cut-off, suggesting potential utility as part of a multi-marker profile, although stand-alone performance was modest. The ROC curves for all elements are illustrated in Figs. 1 and 2, respectively. These ROC findings should be interpreted as exploratory discrimination metrics rather than validated clinical prediction models. In logistic regression analyses (per 1 SD increase), higher Mo was associated with greater odds of GDM (unadjusted OR 1.71, 95% CI 1.12–2.61; *p* = 0.013; age-adjusted OR 1.86, 95% CI 1.14–3.01; *p* = 0.012). Selenium showed the strongest association (unadjusted OR 2.82, 95% CI 1.62–4.92; *p* < 0.001; age-adjusted OR 2.42, 95% CI 1.33–4.42; *p* = 0.0038). Iron was also associated with GDM (unadjusted OR 2.21, 95% CI 1.35–3.64; *p* = 0.0017; age-adjusted OR 2.16, 95% CI 1.27–3.68; *p* = 0.0046) (Supplementary Table S1).

**Table 6:** ROC analysis of trace and heavy metal concentrations in predicting GDM

Variable	Cut-off	Sensitivity (%)	Specificity (%)	AUC	*p*
Cadmium (Cd)	NA	NA	NA	NA	0.842
Lead (Pb)	NA	NA	NA	NA	0.595
Manganese (Mn)	NA	NA	NA	NA	0.238
Tin (Sn)	NA	NA	NA	NA	0.132
Arsenic (As)	NA	NA	NA	NA	0.380
Molybdenum (Mo)	1.05	80.0	46.9	0.662	0.005
Zinc (Zn)	65.5	74.0	51.0	0.654	0.008
Copper (Cu)	161.5	82.0	52.0	0.681	0.002
Selenium (Se)	46.5	72.0	71.4	0.754	< 0.001
Iron (Fe)	85.5	68.0	65.3	0.714	< 0.001
Chromium (Cr)	NA	NA	NA	0.491	0.878
Vanadium (V)	0.7	68.0	75.5	0.700	0.001

## Discussion

In the present study, we demonstrated that pregnant women with gestational diabetes mellitus (GDM) exhibit significant alterations in redox-active trace elements and endothelial biomarkers compared to healthy controls. Specifically, molybdenum (Mo) concentrations were significantly elevated in the GDM group, while nitric oxide synthase (NOS) activity was markedly reduced. No significant differences were observed in total antioxidant status (TAS), total oxidant status (TOS), or asymmetric dimethylarginine (ADMA). The lack of difference in systemic markers (TAS/TOS) highlights that trace element dysregulation in GDM might act through specific enzymatic pathways (e.g., molybdoenzymes, selenoproteins) rather than causing generalized systemic oxidative damage. These findings suggest that trace element dysregulation in GDM may relate to endothelial NO bioavailability through pathway-specific mechanisms rather than a uniform shift in global oxidative indices. However, the present observational data do not allow us to determine whether these alterations are localized to the endothelium or represent downstream correlates of metabolic dysregulation. These findings indicate that dysregulation of trace element homeostasis, particularly involving Mo, Se, and Fe, may contribute to impaired endothelial nitric oxide (NO) bioavailability and oxidative stress in GDM, supporting a hypothesis-generating “Mo–Se–Fe–NOS signature (observational multi-marker profile).” The term ‘signature’ is employed here to emphasize that the observed alterations represent a collective biochemical pattern reflective of the GDM-related redox environment, rather than a single, isolated marker change.

Our findings are in line with previous reports showing that essential trace elements participate in the pathogenesis of GDM through oxidative stress, mitochondrial dysfunction, and insulin resistance [6–9]. Elevated levels of selenium (Se), copper (Cu), and iron (Fe) have been observed in GDM and correlated with increased lipid peroxidation and impaired antioxidant capacity [9–13]. Selenium acts as a key cofactor for glutathione peroxidase, and both deficiency and excess may lead to oxidative imbalance. Zachariah et al. [15] reported that supraphysiological Se concentrations induce endoplasmic reticulum stress and endothelial apoptosis, reducing NO availability — consistent with our observation of diminished NOS activity despite elevated Se levels.

The novel finding of increased Mo concentrations in GDM provides a new mechanistic link between trace element imbalance and vascular dysfunction. Mo is essential for the catalytic activity of enzymes such as xanthine oxidase, aldehyde oxidase, and sulfite oxidase [12]. However, excessive Mo may enhance the production of superoxide radicals via xanthine oxidase, leading to oxidative stress and reduced NO bioavailability. Zheng et al. [16] demonstrated an inverse association between maternal Mo levels and glucose concentration, suggesting that altered Mo metabolism may represent either a compensatory antioxidant response or a contributing factor to insulin resistance. At the selected cut-off, Mo showed relatively high sensitivity but modest overall discrimination and limited specificity, indicating that it is unlikely to serve as a strong stand-alone screening marker. Instead, Mo may be more informative as part of a multi-element profile that warrants external validation. In our study, elevated Mo coincided with reduced NOS activity, indicating that dysregulated molybdoenzyme function may impair vascular redox homeostasis.

Endothelial dysfunction has been consistently reported as a hallmark of GDM pathophysiology [8, 9, 13, 17]. Previous molecular studies demonstrated reduced endothelial NO synthesis and eNOS uncoupling in GDM placental tissue [13, 18]. Leiva et al. [19] further emphasized that NO bioavailability and oxidative stress are shared pathological features among GDM, preeclampsia, and maternal hypercholesterolemia. We propose that concurrent alterations in Se-, Fe-, and Mo-related redox biology could plausibly contribute to a biochemical milieu associated with reduced NO bioavailability in GDM. Nonetheless, our study demonstrates co-occurrence and correlations rather than a confirmed directional pathway, and mechanistic studies are required to test causality and sequence. This interpretation is biologically plausible given prior evidence that trace element imbalance relates to placental oxidative stress and vascular tone in complicated pregnancies [20].

The present findings also align with recent data demonstrating persistent vascular impairment even after pregnancy in women with a history of GDM. Stanhewicz et al. [21] reported that post-GDM women exhibit sustained microvascular endothelial dysfunction and oxidative stress, supporting the concept that the endothelial alterations we identified may have long-term metabolic consequences. Collectively, these findings suggest that the combination of elevated Mo, Se, and Fe with reduced NOS activity represents an early biochemical signature of vascular stress in GDM. It is important to note that while our study design is cross-sectional, evaluating markers at the time of diagnosis (24–28 weeks), the distinct profile of these redox-active elements suggests they may serve as candidates for earlier screening. Future longitudinal studies are needed to confirm if the elevated Mo, Se, and Fe pattern is detectable in the first trimester, thereby validating their utility as early predictive biomarkers for GDM risk stratification.

The strength of this study lies in its integrated evaluation of multiple trace elements, oxidative markers, and endothelial enzymes within the same cohort. Previous studies often examined individual metals or oxidative stress parameters in isolation [7, 10–24]. By simultaneously quantifying essential and toxic elements and correlating them with NOS and ADMA levels, we identified a composite biomarker pattern reflective of both metabolic and vascular dysfunction. This comprehensive approach enhances the translational value of trace element research in obstetric medicine, providing a more accurate reflection of systemic redox status.

Nevertheless, some limitations should be noted. The study population was moderate in size, and environmental or dietary sources of trace element exposure were not quantified. Although the sample size was moderate, a post-hoc power analysis confirmed that the study achieved a power (1-β) of 0.87 for detecting significant differences in NOS activity and > 0.80 for trace elements. This ensures that the observed alterations in the trace element profile and endothelial markers are statistically robust and reliable despite the sample size constraints. The cross-sectional design limits causal inference. Future longitudinal studies with larger cohorts and multi-omics integration (metallomics, transcriptomics, and metabolomics) could clarify the causal pathways linking trace element dysregulation to GDM. Assessing placental transport mechanisms and metal–protein interactions will also be critical to delineate maternal–fetal transfer dynamics. Machine learning–based multielement modeling, as suggested by Zheng et al. [16], may further refine diagnostic algorithms and risk prediction. Additionally, we did not assess individual antioxidant systems (e.g., glutathione status, GPx/TrxR activity), which could help identify which components drive the global TAS/TOS profile. Future studies integrating specific antioxidant enzymes with metallomics may better delineate element–redox pathway interactions. Placental tissue was not available for this cohort; thus, we could not evaluate placental trace element distribution or the expression of trace element–associated transporters/enzymes and endothelial NO pathway components. Tissue-level validation represents an important next step. Although multivariable and mixture-based approaches (e.g., Bayesian kernel machine regression) may better capture complex multi-metal effects, these methods typically require larger sample sizes for stable estimation. Therefore, we prioritized parsimonious regression models and emphasize that larger, externally validated cohorts are needed to implement advanced mixture modeling. While advanced mixture-based approaches such as Bayesian kernel machine regression (BKMR) could further elucidate complex multi-metal interactions, our current sample size was prioritized for parsimonious modeling; such high-dimensional analyses remain a key goal for future, larger-scale multi-center cohorts.

Key Message: These findings highlight a hypothesis-generating multi-marker profile linking trace element dysregulation with reduced NO bioavailability in GDM. Prospective, longitudinal studies—ideally starting in early pregnancy—are needed to determine whether this profile improves risk stratification beyond established clinical predictors.

## Conclusion

This study demonstrated that alterations in maternal trace element balance, particularly elevated molybdenum (Mo), selenium (Se), and iron (Fe) levels accompanied by reduced nitric oxide synthase (NOS) activity, are associated with oxidative and endothelial stress in gestational diabetes mellitus (GDM). The integration of these findings supports the presence of an observational multi-marker signature (Mo–Se–Fe–NOS) that may reflect an association between trace element dysregulation and reduced NO bioavailability in GDM.

Our results advocate for a multi-marker approach rather than relying on single trace elements. The identification of a combined ‘Mo–Se–Fe–NOS signature’ offers a more integrated perspective on vascular redox homeostasis. A multi-marker approach may be useful for hypothesis generation and future risk modeling; however, its incremental predictive value over established screening strategies requires validation in larger, independent cohorts.

By identifying a distinctive multi-element pattern rather than isolated changes, our results provide both mechanistic and translational insights into how redox-active elements influence endothelial nitric oxide bioavailability and glucose metabolism. These observations highlight the potential value of trace element profiling as an adjunctive research biomarker approach; future longitudinal studies are needed before considering clinical implementation.

Although further longitudinal and multi-omics studies are required to establish causal relationships and clinical thresholds, the present data underscore the importance of maintaining optimal trace element homeostasis during pregnancy. Our findings bridge molecular mechanisms of redox biology with clinical obstetric outcomes, offering a new perspective for early prevention and management strategies in GDM.

## Supplementary Information

Below is the link to the electronic supplementary material.

Supplementary Material 1

## Data Availability

The datasets used and/or analysed during the current study are available from the corresponding author on reasonable request.
