# Supplementary material for: The Mo–Se–Fe–NOS Signature: Linking trace Element Dysregulation to Endothelial Dysfunction in Gestational Diabetes Mellitus
Source: Biol Trace Elem Res. 2026 Feb 21;204(7):5310–8. doi: 10.1007/s12011-026-05033-5 (PMC13319427; doi:10.1007/s12011-026-05033-5)
Supplement: Supplementary file 1 — Supplementary Material 1 [file 12011_2026_5033_MOESM1_ESM.docx]

Supplementary Table S1. Logistic regression models for GDM

| **Predictor (per 1 SD)** | **Unadjusted OR (95% CI)** | **p** | **Age-adjusted OR (95% CI)** | **p** | **N** |
| --- | --- | --- | --- | --- | --- |
| Mo | 1.71 (1.12–2.61) | 0.013 | 1.86 (1.14–3.01) | 0.012 | 100 |
| Se | 2.82 (1.62–4.92) | <0.001 | 2.42 (1.33–4.42) | 0.0038 | 100 |
| Fe | 2.21 (1.35–3.64) | 0.0017 | 2.16 (1.27–3.68) | 0.0046 | 99 |

*Predictors are standardized (per 1 SD increase). Odds ratios (ORs) with 95% confidence intervals (CIs) are reported for unadjusted and age-adjusted models.*
